# Novel Perspectives on *ATP8A2* Regulation: Evidence for Parental Imprinting and Chimeric Transcript Formation

**DOI:** 10.3390/epigenomes10020026

**Published:** 2026-04-06

**Authors:** Abdelhamid Bouramtane, Badreddine Elmakhzen, Amal Ouskri, Mohamed Ahakoud, Laila Bouguenouch, Karim Ouldim, Omar Askander

**Affiliations:** 1 Medical Genetics & Oncogenetics Laboratory, Hassan II University Hospital Center, Sidi Harazem Road, Fez 30070, Morocco; badreddine.elmakhzen@usmba.ac.ma (B.E.); amal.ouskri@usmba.ac.ma (A.O.); mohamed.ahakoud@usmba.ac.ma (M.A.); lbouguenouch@yahoo.com (L.B.); karim.ouldim@usmba.ac.ma (K.O.); 2 Faculty of Medicine, Pharmacy and Dentistry, Sidi Mohamed Ben Abdellah University, Fez 30000, Morocco; 3 Mohammed VI Center for Research and Innovation CM6RI, Rabat 10112, Morocco; oaskander@cm6.ma; 4 Mohammed VI Faculty of Medicine, Mohammed VI University of Health Sciences, Rabat 10112, Morocco

**Keywords:** *ATP8A2*, gene expression, parental imprinting, chimeric transcript

## Abstract

**Background**: Parental imprinting plays a crucial role in epigenetic regulation and is increasingly recognized for its involvement in neurodevelopmental disorders. Although *ATP8A2* is considered a non-imprinted gene; However, the marked phenotypic variability observed across related disorders suggests that additional regulatory layers may influence its expression. **Methods**: We investigated the imprinting-like status of *ATP8A2* through functional analyses of a splicing variant (c.1580-3C>G) identified in a patient diagnosed with Cerebellar Ataxia, Mental Retardation, and Disequilibrium syndrome type 4 (CAMRQ4). Sanger sequencing was used to assess allelic expression and identify aberrant transcripts. **Results**: Our analyses revealed an allelic expression imbalance suggestive of parental imprinting of *ATP8A2*. Moreover, Sanger sequencing led to the identification of a novel *ATP8A2*–*RAB3GAP2* chimeric transcript, pointing to a previously unreported transcriptional event, the functional relevance of which remains to be determined. **Conclusions**: These findings indicate that *ATP8A2* may be subject to imprinting-like regulation and involved in atypical splicing events with unknown significance. This highlights the need for further investigation into the epigenetic and transcriptional complexity of *ATP8A2*-related neurodevelopmental disorders.

## 1. Introduction

Gene expression is finely regulated by a multitude of epigenetic mechanisms that orchestrate development, cellular differentiation, and the homeostasis of complex organisms. Among these mechanisms, parental imprinting is a fascinating phenomenon in which certain genes are expressed only from the allele inherited from one parent, while the other allele is silenced by specific epigenetic marks [1]. This monoallelic expression pattern represents a unique form of transcriptional regulation that ensures a delicate balance between maternal and paternal genomic contributions. This process, which affects a limited number of genes in mammals, is essential for embryonic development and neurological function. Its evolutionary conservation across species underscores its biological importance and its disruption can lead to profound developmental and metabolic consequences. Its dysregulation is implicated in various pathologies, including imprinting disorders (such as Prader–Willi and Angelman syndromes), neurodevelopmental disorders, and certain cancers [2].

Parental imprinting is primarily based on stable epigenetic modifications, such as DNA methylation and histone modifications, which either prevent or promote the expression of an allele depending on its parental origin [3]. These epigenetic signatures are established during gametogenesis, maintained through embryonic cell divisions, and erased and reset in the germline to ensure proper inheritance patterns. Imprinted genes are often clustered in imprinting domains, regulated by imprinting control regions (ICRs), which coordinate these epigenetic marks [4]. Such ICRs act as molecular switches controlling the expression of entire gene networks, including protein-coding genes and long noncoding RNAs that frequently interact in cis. While several of these genes have been well characterized, it is likely that others remain undiscovered. Identifying new imprinted genes is a major challenge for advancing our understanding of developmental epigenetics and human diseases. Recent advances in transcriptomics, methylome mapping, and single-cell sequencing have revealed that imprinting effects may be far more dynamic and tissue-specific than previously assumed [5,6].

In parallel with parental imprinting, another molecular phenomenon has intrigued the scientific community: the formation of chimeric transcripts, resulting from the fusion of RNA molecules transcribed from distinct genes. Unlike classical genomic fusions, which involve chromosomal rearrangements, chimeric transcripts can arise at the transcriptional level through a mechanism known as intergenic transcription or trans-splicing between adjacent genes, or through post-transcriptional events involving mRNA rearrangement [5,6,7]. This process illustrates the remarkable flexibility of the eukaryotic transcriptome and the complexity of RNA maturation events that can reshape gene boundaries.

Initially regarded as anomalies specific to cancer cells, chimeric transcripts are now emerging as potentially physiological and functional elements in various biological contexts. They can encode fusion proteins, act as regulatory RNAs, or interfere with canonical gene expression through competition for transcriptional or translational machinery. Several studies suggest that they may play a key role in transcriptome plasticity, protein diversity, and the regulation of genetic networks [8,9]. Some chimeric transcripts have been implicated in various pathologies, particularly in cancer, where they can confer proliferative advantages to tumor cells. However, their role in normal physiological processes remains largely unexplored, and their interaction with other regulatory mechanisms, such as parental imprinting, remains largely unknown [10,11].

*ATP8A2* encodes a neuronal P4-ATPase crucial for maintaining phospholipid asymmetry in axons and synapses; its dysfunction causes severe neurodevelopmental disorders including CAMRQ4 syndrome [12]. The broad variability in neurological severity observed in *ATP8A2*-related cases suggests that additional layers of regulation—beyond DNA sequence alone—may influence phenotypic expression.

Our study was not initially designed to investigate parental imprinting or unconventional transcriptional mechanisms. Rather, it aimed to functionally characterize a splice-site variant (c.1580-3C>G) identified in a patient with a neurodevelopmental phenotype [12]. However, during these analyses, we unexpectedly observed features suggestive of non-canonical regulation of *ATP8A2* expression, including an allelic expression imbalance and the presence of an atypical transcript. These observations led us to hypothesize that *ATP8A2* may be subject to an imprinting-like regulatory mechanism, potentially contributing to variability in gene expression and clinical manifestations. This hypothesis is explored here in a strictly exploratory and hypothesis-generating framework.

## 2. Methods

Our hypothesis emerged within the framework of an experimental study aimed at confirming the pathogenicity of a splicing variant in the *ATP8A2* gene, identified through whole-exome sequencing in a patient presenting with psychomotor delay associated with paraparesis [12]. Whole-exome sequencing was performed as a service by 3billion, Inc. (Seoul, Republic of Korea). Variant annotation and interpretation were conducted using Mobidetails (France) and VarSome (Saphetor S.A., Lausanne, Switzerland), which integrate multiple in silico prediction tools, including splice-site prediction algorithms. The variant NM_016529.6:c.1580-3C>G was predicted to be deleterious by several of these embedded computational predictors.. However, despite converging computational evidence, it remained insufficient to conclusively determine its functional impact. This uncertainty justified the need for direct functional validation to assess its effect on mRNA processing and the downstream consequences on gene function.

**Figure 1:**
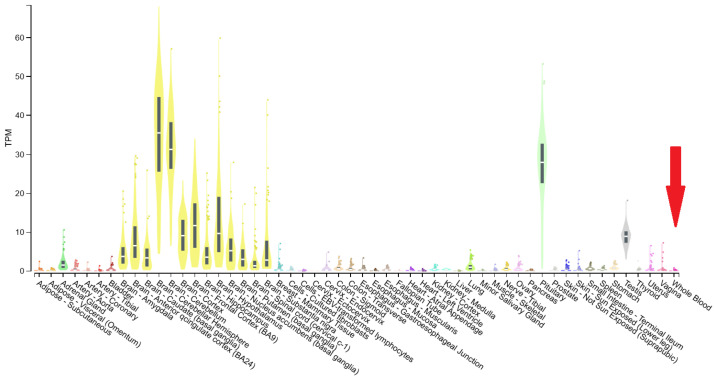
Bulk tissue gene expression for *ATP8A2* [13]. The red arrow indicates the low expression level in whole blood.

To experimentally evaluate the impact of this variant, primers (Table 1) were designed to amplify the cDNA encompassing the exons upstream and downstream of exon 18 using the Primer Designing Tool provided by the National Center for Biotechnology Information (NCBI), National Institutes of Health (NIH, Bethesda, MD, USA). Total RNA was extracted from peripheral blood samples using the PureLink™ Total RNA Blood Purification Kit (Invitrogen, Thermo Fisher Scientific, Waltham, MA, USA), according to the manufacturer’s instructions. cDNA synthesis was performed using the SuperScript™ VILO™ cDNA Synthesis Kit (Invitrogen, Thermo Fisher Scientific, Waltham, MA, USA). PCR amplification was carried out using the DreamTaq™ Green PCR Master Mix (Thermo Fisher Scientific, Waltham, MA, USA). Although *ATP8A2* is known to be weakly expressed in peripheral blood—its expression being primarily restricted to neuronal tissues such as the cerebellum, retina, and spinal cord (Figure 1)—we adopted a two-step nested PCR strategy to enhance sensitivity. This “double PCR” approach, consisting of an initial amplification followed by a second reaction using diluted first-round products as templates, was intended to increase the likelihood of detecting trace transcripts while minimizing background noise and non-specific products.

We anticipated that, if the variant disrupted the canonical acceptor splice site, exon 18 would be skipped, leading to a shorter PCR product relative to the control sample.

**Figure 2:**
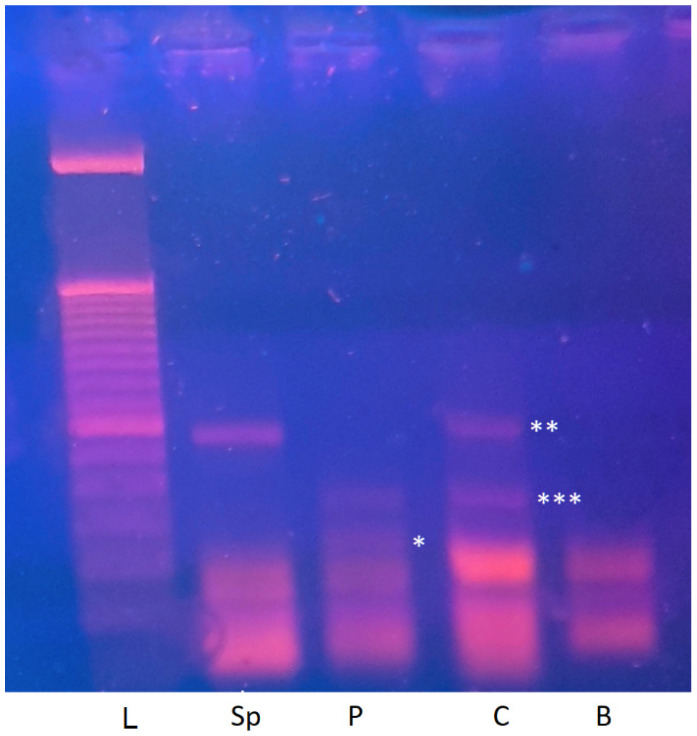
Agarose gel image after migration of PCR products. Samples legend: B (blank), C (control), P (patient), Sp (control sperm), L (ladder 50 bp). Band significance: * abnormal transcript (175 bp), ** predominant canonical transcript (341 bp), *** non-canonical transcript (225 bp) detected in blood (C, P) but absent in sperm (Sp).

Upon agarose gel electrophoresis, the results were revealing. In the control sample, we observed a clear band corresponding to the expected cDNA fragment spanning exons 17–19 (341 bp). Interestingly, an additional band of approximately 225 bp was consistently visible across replicates. This smaller amplicon—subsequently labeled “AB”—did not correspond to any predicted alternative transcript of *ATP8A2* based on current transcriptome annotations. In the patient sample, electrophoresis revealed a predominant shorter band compatible with exon 18 skipping, together with the same 225 bp “AB” band present in the control (Figure 2).

These findings supported two key interpretations. First, the main shorter fragment in the patient confirmed that the variant indeed induced abnormal splicing, justifying its reclassification from a Variant of Uncertain Significance (VUS) to Pathogenic (P) according to ACMG rule PVS1(Null variant). Second, the persistence of the unexplained “AB” band in both samples suggested the existence of an additional, perhaps naturally occurring transcript, independent of the splicing defect. This unexpected result became the turning point of our investigation.

Initially, we hypothesized that the “AB” fragment might represent an unannotated alternative splice isoform. To test this assumption, we conducted an exhaustive in silico exploration. Using the Ensembl genome browser, we verified all known *ATP8A2* transcripts and confirmed the presence of two other protein-coding isoforms differing in their 5′ regions. However, these differences did not involve our region of interest encompassing exon 18, thereby failing to explain the shorter band.

We next examined tissue-specific exon usage through the GTEx database (https://gtexportal.org), which provides large-scale RNA-seq data across multiple human tissues. Expression analysis did not reveal any exon-specific drop or gain in our studied region, suggesting that classical alternative splicing was unlikely. After eliminating possible explanations such as pseudogene interference, non-specific primer binding, or contamination, we began to consider more unconventional mechanisms.

One possibility was that the observed double-band pattern reflected allelic expression differences rather than classical splicing variation. Such differences can occur when one parental allele is preferentially expressed, as seen in imprinted genes (e.g., RB1).

**Figure 3:**
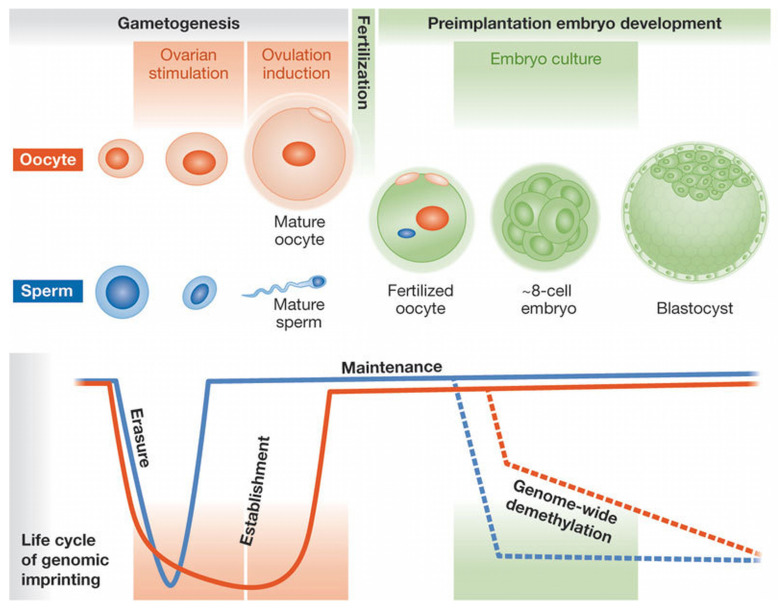
The Life Cycle of Parental Imprinting in Humans [15].

To explore the possibility of parent-of-origin–dependent expression of *ATP8A2*, we included a sperm-derived sample from a healthy adult donor. It should be noted from the outset that mature spermatozoa are transcriptionally inactive and retain a selective RNA repertoire originating from earlier stages of spermatogenesis; thus, sperm RNA reflects the paternal germline transcriptional history rather than active gene expression. RNA was extracted directly from the ejaculate without additional purification steps, an approach justified by the fact that ejaculated semen is overwhelmingly enriched in mature spermatozoa, with minimal contribution from somatic cells [14]. Spermatozoa nonetheless represent a relevant biological context for studies related to imprinting, as paternal epigenetic marks are re-established during spermatogenesis and transmitted at fertilization (Figure 3). Analysis of sperm RNA therefore provides access to transcripts associated with the paternal germline, and in this context, if *ATP8A2* expression were indeed parent-of-origin–dependent, only the paternal transcript would be expected to be detectable in sperm.

Furthermore, to better characterize the molecular nature of the “AB” fragment, we excised it from the gel, purified the DNA using a silica-membrane extraction kit, and subjected it to Sanger sequencing.

## 3. Results

The experiment yielded striking results. Gel electrophoresis showed a single distinct band corresponding to the 341 bp fragment (exons 17–19), identical to the main control band and lacking the 225 bp “AB” band (Figure 2). These observations were consistent with the possibility that the larger transcript could originate from a paternal allele, whereas the shorter “AB” band might reflect a maternally expressed or potentially silenced allele. To address the alternative explanation that this pattern could simply result from tissue-specific expression differences between blood and sperm, we re-examined publicly available GTEx datasets. No marked differences in exon 17–19 coverage across tissues were observed, suggesting that the observed pattern is less likely to be explained solely by tissue-dependent variability and may be compatible with a parent-of-origin–related regulatory effect [13].

**Figure 4:**
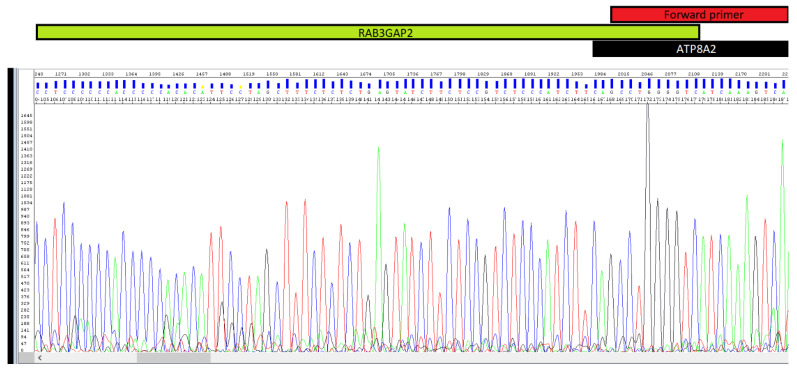
Electropherogram showing the reverse sequence of the chimeric transcript, covering part of the *ATP8A2* gene (where the forward primer binds) and part of the *RAB3GAP2* gene.

**Figure 5:**
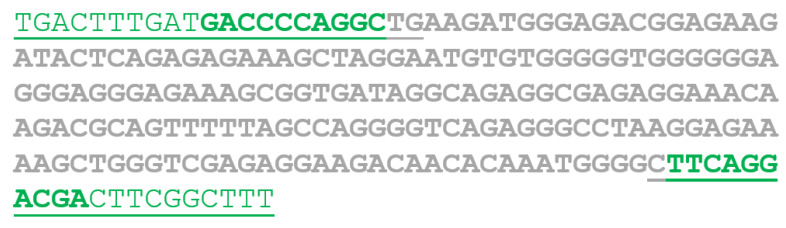
Schematic representation of the AB sequence: The bold sequence originates from the *RAB3GAP2* gene; the underlined sequence originates from the *ATP8A2* gene; the green sequence corresponds to the primer binding sites.

The results of Sanger sequencing were both surprising and illuminating. Contrary to our expectation of identifying a truncated or alternative *ATP8A2* isoform, the sequence alignment revealed that one portion of the fragment matched *ATP8A2*, while the remainder aligned with an entirely different gene located on chromosome 1—*RAB3GAP2* (Figure 4 and Figure 5).

## 4. Discussion

Our findings have potential implications for the understanding of *ATP8A2* regulation and the interpretation of genetic variants. They suggest that epigenetic mechanisms and transcriptional variability may interact to influence neuronal gene expression and related disease processes.

- Parental Imprinting and Potential Regulation of *ATP8A2*:

The observation of a possible parent-of-origin–dependent expression pattern for *ATP8A2* raises questions regarding its regulatory mechanisms. If supported by further studies, these findings would suggest that *ATP8A2* may represent a candidate for inclusion among imprinted genes [16], known for playing a crucial role in neurodevelopment and disease susceptibility. Such an addition would expand our current understanding of imprinting mechanisms beyond the classical loci, suggesting that even genes previously considered biallelically expressed may be partially or contextually imprinted.

Future studies should explore DNA methylation profiles, histone modifications, and regulatory elements at the *ATP8A2* locus to better assess the presence and extent of parent-of-origin–dependent regulation. Should such regulation be supported, further work would be required to determine whether it involves the entire gene, as described for loci such as SNRPN and UBE3A in Prader–Willi and Angelman syndromes [17], or is instead restricted to specific regions or exons, as reported for *RB1* [18].

- Chimeric Transcripts: A Potential Layer of Non-Canonical Regulation

The identification of an *ATP8A2*–*RAB3GAP2* chimeric transcript raises the possibility that non-canonical transcriptional events may contribute to the regulatory landscape of *ATP8A2*. Such cross-chromosomal chimeric RNAs are uncommon and are generally attributed to trans-splicing or post-transcriptional RNA rearrangement mechanisms [10,11]. Although chimeric transcripts have been extensively described in oncological contexts, their occurrence and potential relevance in normal tissues remain poorly understood. In most reported cases, physiologically detected chimeric RNAs involve neighboring genes [10,11]. The *ATP8A2*–*RAB3GAP2* fusion is therefore unusual, as the two genes reside on different chromosomes. Consistently, no corresponding entry was identified in the ChiTaRS/*ChimerDB* [19], suggesting that this transcript is not a recurrent or widely characterized event.

The biological significance of this chimeric transcript remains uncertain. Several non-mutually exclusive interpretations may be considered. The fusion transcript may represent a low-frequency transcriptional byproduct without direct functional consequences. Alternatively, it could exert regulatory effects at the RNA level, potentially influencing transcript stability, subcellular localization, or interactions with RNA-binding proteins or microRNAs targeting *ATP8A2* or *RAB3GAP2*. Given the established role of *RAB3GAP2* in synaptic vesicle trafficking and neuronal signaling [20], such regulatory interference could, in theory, contribute to neurodevelopmental phenotypes; however, no functional evidence currently supports this hypothesis.

Importantly, the absence of known direct protein–protein interactions between *ATP8A2* and *RAB3GAP2* in the STRING database [21], further supports the notion that this fusion does not reflect a canonical functional partnership. Additional investigations using quantitative and allele-specific transcriptomic approaches, as well as analyses across different tissues, developmental stages, and pathological contexts, will be required to determine whether this chimeric RNA reflects a rare but genuine regulatory mechanism or an incidental outcome of RNA processing.

- Clinical and Diagnostic Implications:

The findings reported in this study, although exploratory, raise important considerations regarding the interpretation of *ATP8A2*-related variants and the molecular mechanisms underlying phenotypic variability. While these observations do not establish definitive clinical rules, they may nonetheless provide a conceptual framework for rethinking certain unresolved aspects of *ATP8A2*-associated disorders.

1. Variant Interpretation Based on Parental Origin

If parental imprinting is confirmed, the pathogenicity of *ATP8A2* variants may depend on their parental origin, necessitating a reassessment of genetic counseling strategies. In practical terms, this would imply that a pathogenic variant carried on the transcriptionally active parental allele (e.g., paternal) could be sufficient to produce a clinical phenotype, even in the heterozygous state, whereas the same variant on a transcriptionally silenced allele might remain clinically silent. Accordingly, variant interpretation should not rely solely on zygosity but should also take into account epigenetic context and parent-of-origin effects. Similarly, this hypothesis would have a direct impact on the diagnosis of CAMRQ4 syndrome (Cerebellar Ataxia, Mental Retardation, and Quadrupedal Gait type 4 syndrome). Exome sequencing in patients with a suggestive clinical presentation but previously classified as negative due to the absence of biallelic mutations should be reanalyzed. A heterozygous variant on the functional allele could be sufficient for diagnosis, preventing misinterpretations and improving patient management.

2. Functional Interaction and Metabolic Regulation

The ATP8A2–RAB3GAP2 chimeric transcript raises the possibility of a previously unrecognized molecular link between two genes whose disruption leads to distinct neurodevelopmental disorders. Pathogenic variants in ATP8A2 cause CAMRQ4, whereas biallelic variants in RAB3GAP2 are responsible for Martsolf syndrome. The existence of a shared transcript, even if rare, may prompt consideration of overlapping or atypical phenotypes in patients with unresolved neurodevelopmental presentations [20].

Although no evidence currently supports the production of a functional fusion protein, the chimera could theoretically interfere with the expression or regulation of canonical ATP8A2 or RAB3GAP2 transcripts. Such interference might contribute to phenotypic variability, incomplete genotype–phenotype correlations, or unexplained clinical severity in selected cases. This hypothesis is particularly relevant given the central role of both genes in neuronal polarity, vesicle trafficking, and synaptic function [22,23].

3. Future Therapeutic Considerations

A deeper understanding of the epigenetic regulation of *ATP8A2* could pave the way for targeted therapies, such as the use of epigenetic modulators to modify imprinting patterns or RNA-based approaches to counteract the effects of aberrant splicing [24,25]. Pharmacologic demethylation agents, small-molecule histone modifiers, or allele-specific RNA interference could, in theory, restore normal expression balance between parental alleles [26]. Similarly, antisense oligonucleotides might be designed to inhibit the formation of aberrant chimeric transcripts, representing a precision-medicine approach for *ATP8A2*-related disorders. Ultimately, understanding imprinting mechanisms in *ATP8A2* is not merely of academic interest—it may open a therapeutic window for modulating gene expression and alleviating neurological dysfunction in affected individuals.

- Challenges in Experimental Validation and Alternative Approaches

Having uncovered observations suggestive of imprinting-like regulation, we recognized that formal validation of the imprinting hypothesis would require transcriptomic and methylation-specific approaches. However, given our limited access to appropriate techniques, we propose an alternative validation framework based on targeted experimental models. Specifically, this approach would involve performing the same PCR assay on cDNA derived from: (1) oocytes, in which a reciprocal pattern relative to sperm—namely, the presence of the AB band—might be anticipated; (2) blood samples from patients carrying a heterozygous deletion encompassing the entire *ATP8A2* locus, a context in which expression from a single parental allele would be expected, resulting in a single-band profile; and (3) individuals with uniparental disomy (UPD) of chromosome 13, who would be expected to exhibit expression from only one parental origin.

Such experiments would provide decisive evidence for or against imprinting of *ATP8A2* by revealing whether expression depends on parental origin. Unfortunately, ethical and logistical constraints—particularly the limited availability of oocytes and UPD samples—prevented immediate implementation of these tests. Consequently, our investigation had to be temporarily suspended. Nevertheless, the methodological framework established here could readily guide future research efforts.

From a broader perspective, this study underscores the power of variant-driven investigations to uncover unforeseen biological mechanisms. What began as a routine functional assay for a suspected splicing defect evolved into an exploration of imprinting and intergenic transcriptional complexity. We thus propose that other researchers encountering similar unexplained electrophoretic bands during variant analysis should consider the possibility of imprinting or chimeric transcript formation rather than dismissing such findings as mere artifacts.

We hope that a future researcher will be able to validate this hypothesis based on this work, potentially using advanced methodologies such as allele-specific RNA-seq, DNA methylation profiling, phased SNP-based allele-specific expression analyses, or single-cell transcriptomics to capture rare chimeric or monoallelic events with higher resolution. The integration of these high-throughput technologies would not only clarify *ATP8A2*’s regulatory landscape but could also reveal general principles governing gene expression at the interface of imprinting and RNA processing.

## 5. Methodological Limitations

While this study provides intriguing observations regarding *ATP8A2* transcriptional regulation, several important limitations must be acknowledged and carefully considered when interpreting the results.

### 5.1. Absence of Direct Allele-Specific Expression Analysis

The main limitation of this work is the lack of formal allele-specific expression analysis. Canonical demonstration of genomic imprinting requires the identification of parent-of-origin–specific expression at the allelic level, ideally through RNA sequencing or next-generation bisulfite sequencing. In the present study, these techniques were not available. In addition, the reliance on PCR-based approaches, which are qualitative in nature and susceptible to amplification bias, does not allow quantitative or allele-specific assessment of transcript expression. Consequently, we cannot determine whether the observed expression imbalance is driven by true imprinting mechanisms or by alternative epigenetic, transcriptional, or technical factors.

### 5.2. Interpretation Constraints Related to Sperm RNA Analysis

Although sperm RNA was used as a proxy to explore potential paternal allele expression, it is well established that mature spermatozoa are transcriptionally inactive and contain a highly selective, atypical, and functionally specialized RNA repertoire. The absence of the shorter transcript in sperm RNA therefore cannot be interpreted as definitive evidence of maternal silencing or paternal-specific expression in somatic tissues. Sperm RNA findings should be considered supportive but indirect, and they must be interpreted with caution.

### 5.3. Tissue-Specific and Low-Abundance Expression of ATP8A2

*ATP8A2* is predominantly expressed in neuronal tissues, whereas our functional experiments were performed using peripheral blood RNA, where expression levels are low. Despite the use of nested PCR to enhance sensitivity, this raises the possibility that stochastic monoallelic expression, tissue-specific regulation, or low-abundance transcriptional artifacts could contribute to the observed banding patterns. Therefore, extrapolation of these findings to neuronal tissues should be made cautiously.

### 5.4. Functional Significance of the Chimeric Transcript Remains Unresolved

Although the *ATP8A2*–*RAB3GAP2* chimeric transcript was reproducibly detected and validated by Sanger sequencing, its biological relevance remains unknown. We did not assess its expression level, stability, tissue specificity, or translational potential. It is therefore unclear whether this transcript represents a functional regulatory RNA, a rare physiological trans-splicing event, or a low-frequency transcriptional byproduct without direct functional consequences.

### 5.5. Hypothesis-Generating Nature of the Study

Finally, it is important to emphasize that this work is primarily hypothesis-generating. The observations reported here emerged unexpectedly during functional validation of a splice-site variant and were not the result of a study specifically designed to investigate imprinting or trans-splicing mechanisms. As such, the conclusions should be interpreted as preliminary and intended to stimulate further investigation rather than to establish definitive regulatory models.

## 6. Conclusions and Future Perspectives

Our study provides the first exploratory evidence suggesting that *ATP8A2* may be subject to an unknown imprinting mechanism and that it can form a chimeric transcript with *RAB3GAP2*. While our findings raise intriguing questions, they also highlight the need for further investigations. Future research should focus on:

- Epigenetic characterization of the *ATP8A2* locus to determine its imprinting status.
- Functional analysis of the *ATP8A2-RAB3GAP2* chimeric transcript to assess its potential impact on neuronal function.
- Expanded genetic screening in patients with *ATP8A2*-related disorders to explore whether imprinting influences disease expression.

Ultimately, this work highlights the complexity of gene regulation mechanisms and their profound implications in fundamental biology and clinical genetics.

## Figures and Tables

**Table 1:** Primers used in PCR on cDNA.

Forward	Reverse	Temperature
TGACTTTGATGACCCCAGGC	AAAGCCGAAGTCGTCCTGAA	62°

## Data Availability

No additional datasets were generated or deposited in external repositories. Any additional clarifications can be provided upon reasonable request to the corresponding author.
